# Transient Receptor Potential Melastatin 1: A Hair Cell Transduction Channel Candidate

**DOI:** 10.1371/journal.pone.0077213

**Published:** 2013-10-11

**Authors:** John Gerka-Stuyt, Adrian Au, Neal S. Peachey, Kumar N. Alagramam

**Affiliations:** 1 Otolaryngology Head and Neck Surgery, University Hospitals Case Medical Center, Case Western Reserve University, Cleveland, Ohio, United States of America; 2 Research Service, Louis Stokes Cleveland VA Medical Center, Cleveland, Ohio, United States of America; 3 Cole Eye Institute, Cleveland Clinic, Cleveland, Ohio, United States of America; 4 Department of Ophthalmology, Cleveland Clinic Lerner College of Medicine of Case Western Reserve University, Cleveland, Ohio, United States of America; University of Houston, United States of America

## Abstract

Sound and head movements are perceived through sensory hair cells in the inner ear. Mounting evidence indicates that this process is initiated by the opening of mechanically sensitive calcium-permeable channels, also referred to as the mechanoelectrical transducer (MET) channels, reported to be around the tips of all but the tallest stereocilia. However, the identity of MET channel remains elusive. Literature suggests that the MET channel is a non-selective cation channel with a high Ca^2+^ permeability and ∼100 picosiemens conductance. These characteristics make members of the transient receptor potential (TRP) superfamily likely candidates for this role. One of these candidates is the transient receptor potential melastatin 1 protein (TRPM1), which is expressed in various cells types within the cochlea of the mouse including the hair cells. Recent studies demonstrate that mutations in the *TRPM1* gene underlie the inherited retinal disease complete congenital stationary night blindness in humans and depolarizing bipolar cell dysfunction in the mouse retina, but auditory function was not assessed. Here we investigate the role of *Trpm1* in hearing and as a possible hair cell MET channel using mice homozygous for the null allele of *Trpm1* (*Trpm1^−/−^*) or a missense mutation in the pore domain of TRPM1 (*Trpm1^tvrm27/tvrm27^*). Hearing thresholds were evaluated in adult (4–5 months old) mice with auditory-evoked brain stem responses. Our data shows no statistically significant difference in hearing thresholds in *Trpm1^−/−^* or *Trpm1^tvrm27/tvrm27^* mutants compared to littermate controls. Further, none of the mutant mice showed any sign of balance disorder, such as head bobbing or circling. These data suggest that TRPM1 is not essential for development of hearing or balance and it is unlikely that TRPM1 is a component of the hair cell MET channel.

## Introduction

Hearing functions depend on specialized mechanoreceptors of the inner ear called hair cells that convert sound vibrations into electrical stimuli. The mechanotransduction function of hair cells occurs within the set of stereocilia on each hair cell. The hair cell stereocilia are coupled to one another by intercilliary links including tip links, which are protein structures that connect the tip of each stereocilium to the side of its tallest neighboring stereocilium [1], [2]. With an excitatory stimulus, the hair bundle is deflected towards the tallest stereocilia and the tip links are tensioned and are thought to directly pull open MET channels located at their lower ends [3]. However, the identity of this MET channel remains a mystery. All evidence reported in the literature suggests that the transduction channel is a non-selective cation channel with a high Ca^2+^ permeability and ∼100 picosiemens conductance [4], [5], [6]. These properties make members of the transient receptor potential (TRP) superfamily likely candidates for this role.

TRP channels are tetramers comprised of six transmembrane polypeptide subunits that form permeable ion channels. TRP channels play prominent roles in calcium signaling and are ubiquitously expressed and participate in the perception of a wide variety of sensory stimuli including taste (umami, sweet and bitter), temperature and light [5], [7]. The TRP family can be divided into 7 subgroups: TRPM (melastatin), TRPC (canonical), TRPV (vanilloid), TRPP (polycystin), TRPA (ankyrin), TRPML (mucolipin) and TRPN (NOMPC-like) [5]; some of which have been proposed and tested as hair cell transduction channels [8], [9]. For example, it was reported that TRPA1 is expressed in the stereocilia and inhibition of TRPA1 protein expression in zebrafish and mouse inner ears diminished transduction currents and uptake of channel-permeant fluorescent dye. This suggests that TPRA1 plays a crucial role in the transduction channel [9]. However, a subsequent study by Kwan et al. in 2006 reported that TRPA1 was not essential for hair-cell transduction [10]. They found no evidence of hearing loss or balance disorder in mice lacking TRPA1 expression. Despite these findings, members of the TRP family of proteins continue to be attractive candidates for hair cell MET channel.

Another potential candidate from the TRP family is the transient receptor potential channel melastatin 1 (TRPM1). The TRPM family contains eight channels expressed in the stria vascularis, organ of Corti, outer and spiral ganglion cells [11], and TRPM1 was localized to several inner ear structures (stria vascularis, spiral prominence and spiral ligament, outer hair cells, inner hair cells, cochlear supporting cells, spiral ganglion cells, vestibular hair cells, vestibular dark cells, and in vestibular ganglion cells) [11].

In humans, a mutation in *TRPM1* causes the complete form of congenital stationary night blindness (cCSNB), an autosomal recessive disease that is characterized by depolarizing bipolar cell (DBC) dysfunction [12], [13], [14]. Mice homozygous for the null allele of *Trpm1* (*Trpm1^−/−^*) lacked the b-wave of the electroretinogram (ERG) [15], [16], [17] a component which reflects DBC function in the normal retina. A new mouse mutant, *Trpm1^tvrm27^*, was recently identified in a chemical mutagenesis screen based on a similar ERG b-wave reduction [18]. *Trpm1^tvrm27/tvrm27^* mutants showed an eye phenotype consistent with that reported for *Trpm1^−/−^* mice with one important distinction: *Trpm1^tvrm27/tvrm27^* mice retain expression of the TRPM1 protein on the dendritic tips of DBCs [18]. These reports encouraged us to investigate the role of TRPM1 in hearing and possibly hair cell MET function by using these *Trpm1* mouse mutants.

## Materials and Methods

### Mice

All mice were cared for and treated in accordance to the Institutional Animal Care and Use Committee of Case Western Reserve University (CWRU). *Trpm1^tm1Lex/tm1Lex^*, hereafter referred to as *Trpm1* ^−/−^ mice, were generated by Lexicon Genetics and acquired from the European Mouse Mutant Archive (www.emmanet.org). *Trpm1^tvrm27/tvrm27^* mice were derived from a mutagenesis program [19]. The point mutation *Trpm1^tvrm27^* changes a highly conserved alanine at position 1068 to threonine (p.A1068T) [18]. Four to five month old *Trpm1* ^−/−^ and *Trpm1^tvrm27/tvrm27^* mice were tested. The genotype of each mouse used in this study was confirmed by PCR (for the knockout and sibling controls) or DNA sequence analysis (for the point mutation *tvrm27*).

### Ethics Statement

This study was carried out in strict accordance with the recommendations in the Guide for the Care and Use of Laboratory Animals of the National Institutes of Health and animal welfare guidelines at CWRU, USA. The protocol was approved by the Institutional Animal Care and Use Committee at CWRU (Protocol Number: 2012-0068).

### Electrophysiological Testing

To evaluate hearing, auditory-evoked brain stem response (ABRs) was recorded following presentation of pure tone stimulus to the mouse ear at frequencies of 8, 16 and 32 kHz in accordance with previous descriptions [20]. Briefly, mice were anesthetized with intraperitoneal injection of rodent cocktail (ketamine 40 mg/kg, xylazine 5 mg/kg, and acepromazine 1 mg/kg) at doses of 40, 5, and 1 mg/kg. Once anesthetized, the body temperature was maintained at 37°C by placing the mouse on a homeothermic pad (Harvard Apparatus) setup inside a soundproof chamber. ABRs were recorded using a SmartEP system from Intelligent Hearing Systems (Miami, FL). Platinum needle electrodes were inserted (subdermal) at the vertex and ventrolaterally to the right ear and left ear. To test hearing, mice were presented with 8, 16 or 32 kHz pure tone stimuli at a stimulus intensity starting at 100 decibel sound pressure level (dB SPL) and decrementing in 5 dB steps until the lowest intensity that evoked a reproducible ABR waveform. The stimulus was presented for 100 millisecond duration and for at least 500 sweeps to the ear through high-frequency transducers (a closed system). The left and right ears were tested separately and the thresholds were averaged.

### Statistical Analysis

The data were collected in Excel® (Microsoft, Redmond, Washington); statistical analysis was performed using GraphPad Prism® (GraphPad software, San Diego, California). A Mann-Whitney test was used to compare thresholds from *Trpm1* ^−/−^ or *Trpm1^tvrm27/tvrm27^* mice to controls (*Trpm1* ^+/−^ or *Trpm1* ^+/+^ mice).

### Genotyping

*Trpm1* ^−/−^ allele: Details of the targeted deletion of *Trpm1* was reported previously [15]. The genotype of the mice was confirmed by allele specific PCR product. Genomic DNA (∼500 ng/reaction) obtained from tail biopsies were amplified using 1 µM of each primer (KA1215: GGCATGTGTAGCTACCACAG and KA1216: GCATAGTCCATGGACCTAGC or KA1215 and KA1217: GCAGCGCATCGCCTTCTATC), platinum Taq polymerase and PCR buffer as suggested by the manufacturer (Life Technologies, CA). The reaction mix was cycled 35 times at 94°C, 20 seconds, 55°C, 20 seconds and 72°C, 60 seconds. The PCR products were resolved on 2% agarose gel. An 840 base pair fragment for the wild-type and 280 base pair fragment for the knockout allele are expected.

*Trpm1^tvrm27^* allele: The genotype of the *Trpm1^tvrm27^*mice was confirmed by amplification of the target sequence by PCR (primers used KA1220: ATTCAGGGAGTGCTTGGTTG and KA1221: GGTATCTGCCACCCTCTCAG) followed by DNA sequence analysis. Genomic DNA (∼500 ng/reaction) obtained from tail biopsies were amplified using Platinum Taq DNA polymerase and reaction buffer supplied by the manufacturer (Invitrogen, CA). PCR was carried out for 35 cycles (94°C for 20 seconds, 55°C for 30 seconds, and 72°C for 30 seconds). The 639 base pair PCR product was resolved by agarose gel electrophoresis (2% agarose gel). Primer KA1220: ATTCAGGGAGTGCTTGGTTG was used to sequence the PCR product.

### RT-PCR

To determine if *Trpm1* mRNA is expressed in the cochlea of *Trpm1^−/−^* mice, RT-PCR analysis was carried out as described previously [21]. Briefly, total RNA from control and mutant mice cochlea were extracted using Trizol reagent (Life Technologies, CA) followed by DNase I treatment (Qiagen, USA). Total RNA was purified using RNeasy Mini Kit reagents and protocol, as described by the manufacturer (Qiagen, USA). First strand cDNA was synthesized by reverse transcription (Life Technologies, CA) and the expression of *Trpm1* message was detected by amplification of the target cDNA by PCR (primers used KA1223: ACCTCATGGTGAAGGACTGG and KA1224: TTCCTTTGAGCAAGGCAGTT). PCR amplification of *β-actin* cDNA (primers used, KA745: GGGAATGGGTCAGAAGGACT. reverse primer KA746: ACATCTGCTGGAAGGTGGAC) from each RT reaction served as a control for RNA (quality) and RT-PCR protocol. RNA isolated from C57BL/6J (B6) mice cochlea served as a reference for the expected PCR product size for *Trpm1* and *β-actin* from a wild-type tissue. PCR was carried out for 35 cycles (94°C for 20 seconds, 55–60°C for 20 seconds, and 72°C for 60 seconds). The PCR products (847 base pairs for *Trpm1* and 932 base pairs for *beta-actin*) were resolved using agarose gel (2%) electrophoresis.

## Results and Discussion

**Figure 1:**
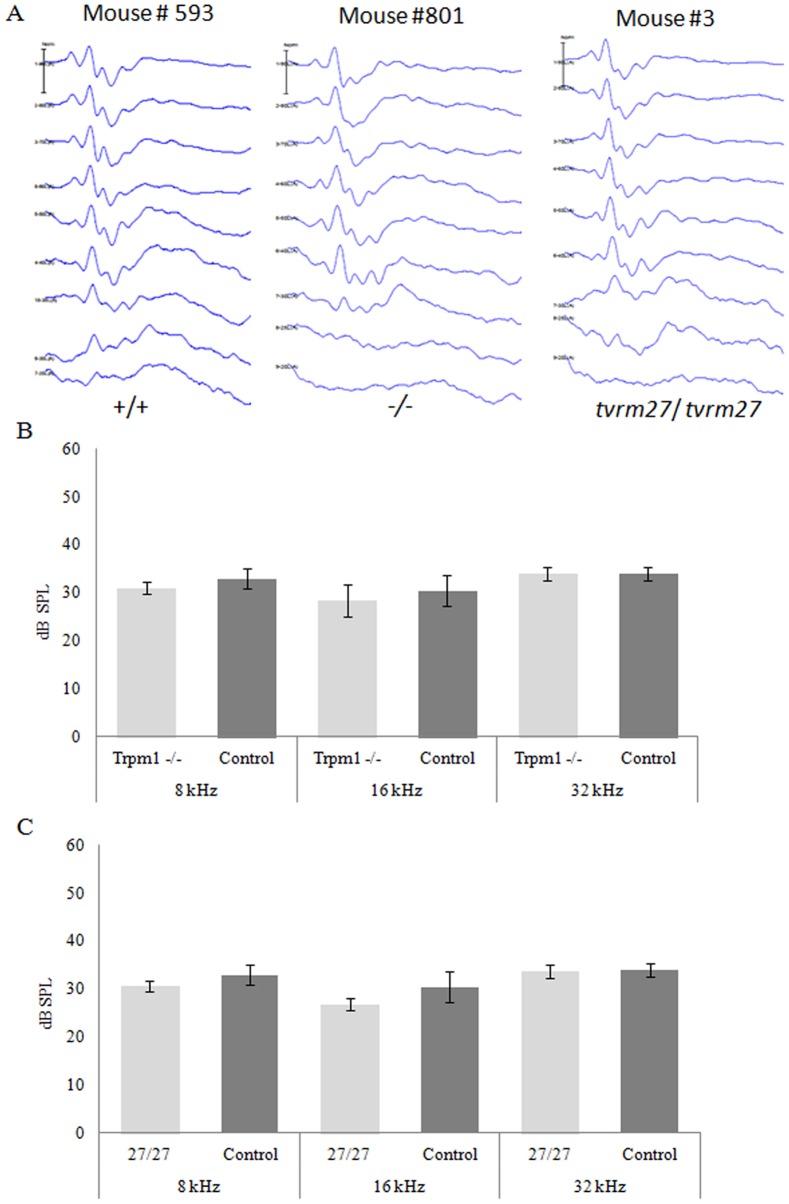
ABR analysis of auditory function in *Trpm1* mutant mice. A. Representative ABR waveforms in response to 16-type (+/+, # 593), *Trpm1^−/−^* (−/−, # 801) and *Trpm1^tvrm27/tvrm27^* (*tvrm27/tvrm27*, # 3) mice. B. Distribution of hearing thresholds of *Trpm1^−/−^* mice (n = 5) compared to controls (*Trpm1^+/+^*or *Trpm1^+/−^* ) (n = 5). Stimulus type: 8 kHz (*P* = 0.11), 16 kHz (*P* = 0.21) and 32 kHz (*P* = 0.5). No statistically significant differences observed between the control and mutants; error bars represent the standard deviation. C. Hearing thresholds of *Trpm1^tvrm27/tvrm27^* (n = 4) mice vs. controls (*Trpm1^+/+^* and *Trpm1^+/−^*) (n = 5) when presented with frequencies of 8 kHz (*P* = 0.10), 16 kHz (*P* = 0.06) and 32 kHz (*P* = 0.64). No appreciable differences in auditory function were observed between *Trpm1* mutants and controls.

**Figure 2:**
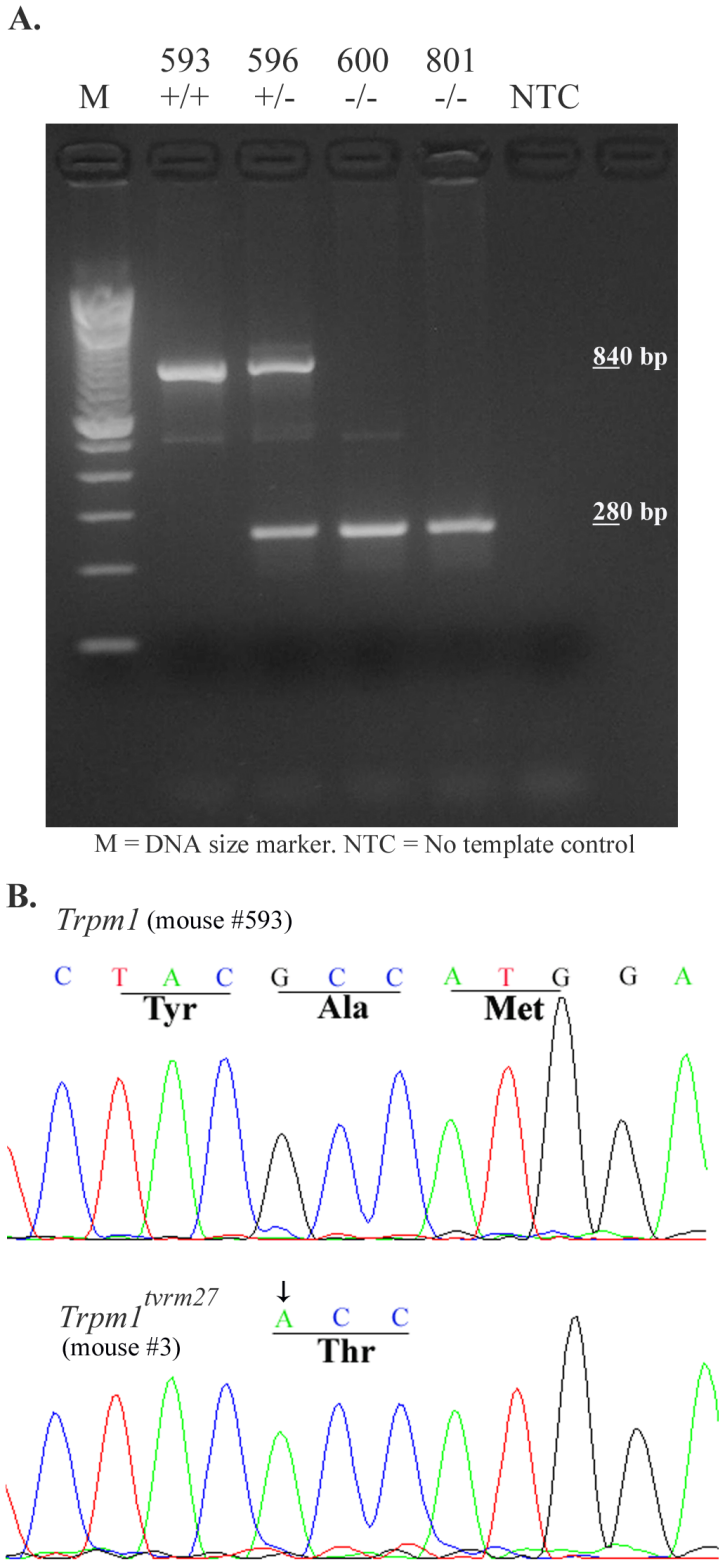
Confirmation of genotype. After terminal ABR recording, genomic DNA isolated from tail biopsies the target sequence was analyzed by PCR or DNA sequencing. Data representative of the genotype analysis is presented here. A. All mice, identified by number above the agarose gel, displayed normal hearing thresholds. Mouse 593 carried wild-type (+/+) allele of *Trpm1*; mice 600 and 801 carried only the *Trpm1^−/−^* (−/−) allele. The 840 and 280 base pair (bp) bands indicate the wild-type and knockout *Trpm1* alleles, respectively. M = DNA size marker (100 base pair size DNA ladder from Invitrogen, CA) B. Chromatogram from DNA sequence analysis of wild-type mouse (#593) and *Trpm1^tvrm27/tvrm27^* mouse (#3). Sequence analysis shows that mouse #3, which displayed normal ABR (Fig. 1A), harbors the G-to-A point mutation in exon 23 that changes the highly conserved amino acid alanine (Ala) at position 1068 to threonine (Thr) in TRPM1.

**Figure 3:**
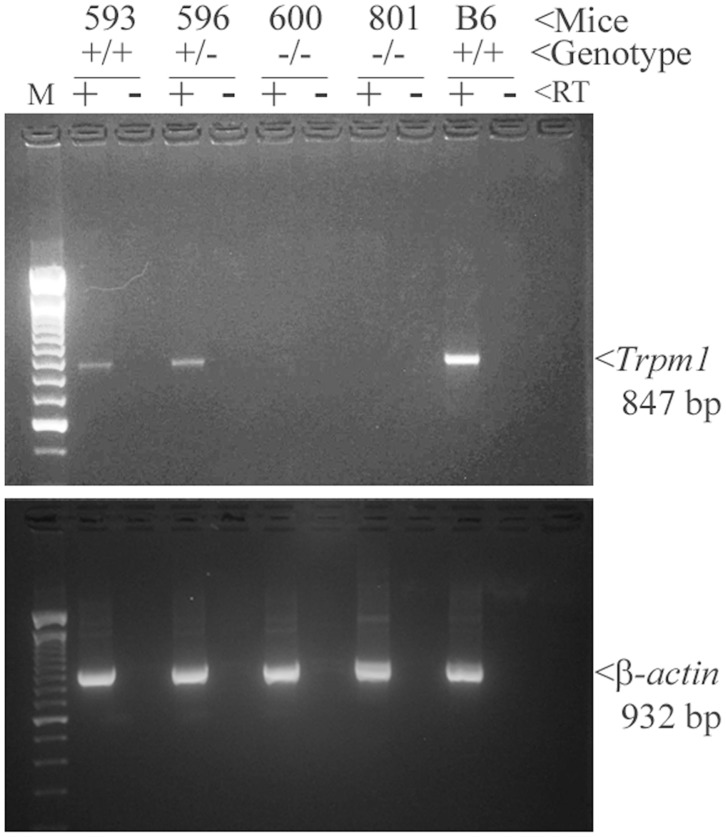
Confirmation of loss of *Trpm1* mRNA expression in the cochlea of *Trpm1^−/−^* mice. Top panel: RT-PCR analysis confirmed loss of *Trpm1* mRNA expression in mice homozygous for the knockout allele (−/−) compared to heterozygotes (+/−) or mice homozygous for the wild-type allele (+/+). Bottom panel: Detection of *beta-actin* mRNA expression in (from the top panel) served as positive control for the presence of RNA in each sample/lane and RT-PCR technique used. C57BL/6J (B6) lane serves as a reference for the expected RT-PCR product size for *Trpm1* and *beta-actin* from wild-type tissue.

Physiological findings associated with mutations in *TRPM1* and the fact that TRPM1 is expressed in hair cells [11] suggests a possible role for TRPM1 in hearing physiology. To test this hypothesis we evaluated auditory function in mice carrying a null mutation or a point mutation in *Trpm1.* Figure 1 (A, B & C) summarizes the ABR results obtained using pure tone stimuli (8, 16, 32 kHz). We saw no difference in hearing sensitivity between *Trpm1* ^−/−^ or *Trpm1^tvrm27/tvrm27^* mice and age-matched controls. The ABR waveforms recorded from the *Trpm1* mutants were comparable to those obtained from control mice. Figure 1A shows representative waveforms from all three genotypes in response to 16 kHz pure tone stimulus: no signs of delayed peak latency and/or reduced peak amplitudes were discernible in the ABR recordings from the *Trpm1* mutants, and ABR thresholds did not differ from control at any frequency examined (Fig. 1B and C). The genotype of mice used to obtain ABR thresholds was confirmed by PCR for the *Trpm1* ^−/−^ allele (Fig. 2A) or DNA sequence analysis for the *Trpm1^tvrm27^*point mutation (Fig. 2B). We confirmed the loss of *Trpm1* mRNA expression in whole cochlear tissue the *Trpm1* ^−/−^ mouse (Fig. 3). The data presented here clearly demonstrate that loss of *Trpm1* expression does not affect hearing in mice. *Trpm1* ^−/−^ mutants did not show any sign of vestibular dysfunction, such as circling behavior or head bobbing (data not shown).

Maintenance of normal hearing and balance function in *Trpm1^−/−^* mice indicates that TRPM1 is not essential for the development of inner ear (vestibular and cochlear) structure or function. More specifically, adult mice display normal inner ear function indicating that hair cell MET function in the mouse inner ear is not dependent on TRPM1. Mice carrying a missense mutation in the pore domain of TRPM1, where the highly conserved alanine at position 1068 is converted to threonine (p.A1068T), also displayed no inner ear phenotype: The *Trpm1^tvrm27/tvrm27^* mice (n = 4) showed hearing thresholds in the normal range (Fig. 1C) and no sign of vestibular dysfunction.

It should be noted that both *Trpm1^−/−^* and *Trpm1^tvrm27/tvrm27^* mice have DBC dysfunction as described in previous ERG and patch clamp studies [15], [16], [17], [18]. A possible explanation for the lack of an inner ear phenotype in *Trpm1* mutant mice is redundancy of related channel protein in the inner ear. There are eight members of the TRPM family and all are expressed in the inner ear [11], suggesting that one could substitute for the loss of TRPM1. TRPM7 is a good candidate since it is reported to be a mechanosensitive channel in vascular tissue [22]. Proteins outside of the TRP family have been reported as possible candidates for MET channels in hair cells. These include the cyclic nucleotide-gated channel α-3(CNGA3) [23], [24] and transmembrane channel-like (TMC) proteins [25]. The TRP channel literature supports the idea of redundancy. For example, Quick et al. found that TRPC3 and TRPC6, two transient receptor potential similar to TRPM1, are expressed in both sensory neurons and cochlear hair cells [26] and reported that an inner ear phenotype was not observed in the single knockout for TRPC3 or TRPC6. Mice lacking both TRPC3 and TRPC6, however, presented with high frequency hearing loss and vestibular dysfunction, indicating that expression of one channel can mask the deficiency of the other. Mutations in TMC1 is linked to dominant and recessive deafness in humans and mice [27], [28] but normal hair cell mechanosensitvity was reported in mice carrying an in-frame deletion in *Tmc1* [29]. A subsequent report by Kawashima and colleagues, however, showed genetic deletion of *Tmc1* and *Tmc2* eliminates hair cell mechanosensitivity, suggesting that expression of *Tmc2* masked any loss of mechnosensitivity associated with mutation in *Tmc1* [25].

The second possibility for the lack of an auditory phenotype in *Trpm1^−/−^* mice could be compensatory up-regulation of a related channel or heteromeric partner. For example, it has been reported that deletion of *Trpc6* leads to an upregulation of *Trpc3* expression in smooth muscle [30]. Since TRPC3/6 heteromers have been implicated in cochlear hair cell MET function, upregulation of TRPC3 could potentially compensate for the loss of TRPC6 expression.This scenario is less likely to explain lack of ear phenotype in *Trpm1^−/−^* mice. It should be noted that *Trpm1^tvrm27/tvrm27^* mutants show an eye phenotype (loss of function) consistent with that reported for *Trpm1^−/−^* mice with an important difference: *Trpm1^tvrm27/tvrm27^* mice retain expression of the TRPM1 protein on the dendritic tips of DBCs [18]. Similarly, we expect *Trpm1^tvrm27/tvrm27^* mice to retain expression of the mutant TRPM1 protein in hair cells. Since comparable ABR thresholds were obtained in the *Trpm1^−/−^* mutant and the loss of function mutant *Trpm1^tvrm27/tvrm27^*, we believe the knockout mutation in *Trpm1* is less likely to trigger a compensatory response. Further, if TRPM1 mediated its function as a heteromer with a related channel protein, then we would expect to observe loss of hair cell function to some degree in the *Trpm1^tvrm27/tvrm27^* mutants due to a dominant negative effect. Our data suggests that TRPM1 is less likely to function as heteromers in the ear.

The data presented in this report demonstrates that TRPM1 is not essential for development of hearing or balance and it is unlikely that TRPM1 is the mechanotransduction channel in mouse auditory hair cells. This conclusion is consistent with the lack of auditory involvement in patients with cCSNB due to mutations in *TRPM1* [12], [13], [14]. TRPM1 has been linked to signal transduction in DBCs, and presumably acts as the signal transduction channel [17], [31]; TRPM1 also appears to impact coat color [12] and to play a role in melanocyte physiology [32] More studies will be necessary to fully understand the role TRPM1 in the mammalian system. The availability of an allelic series of *Trpm1* mouse mutants with different effects on gene expression and trafficking provides important tools with which to understand TRPM1 function in the body.

### Conclusions

TRPM1 is not essential for the development of hearing or balance in the mouse model and it is unlikely that TRPM1 is the hair cell mechanotransduction channel.
